# P2Y_1_ Receptor Signaling Contributes to High Salt-Induced Priming of the NLRP3 Inflammasome in Retinal Pigment Epithelial Cells

**DOI:** 10.1371/journal.pone.0165653

**Published:** 2016-10-27

**Authors:** Philipp Prager, Margrit Hollborn, Anja Steffen, Peter Wiedemann, Leon Kohen, Andreas Bringmann

**Affiliations:** 1 Department of Ophthalmology and Eye Hospital, University of Leipzig, Leipzig, Germany; 2 Helios Klinikum Aue, Aue, Germany; Eye Hospital, Charité, GERMANY

## Abstract

**Background:**

Systemic hypertension is a risk factor of age-related macular degeneration (AMD), a chronic inflammatory disease. Acute hypertension is caused by increased extracellular osmolarity after intake of dietary salt (NaCl). We determined in cultured human retinal pigment epithelial (RPE) cells whether high extracellular NaCl alters the gene expression of inflammasome-associated proteins, and whether autocrine/paracrine purinergic (P2) receptor signaling contributes to the NaCl-induced NLRP3 gene expression.

**Methodology/Principal Findings:**

Hyperosmolarity was induced by the addition of 100 mM NaCl or sucrose to the culture medium. Gene and protein expression levels were determined with real-time RT-PCR and Western blot analysis, respectively. IL-1β and IL-18 levels were evaluated with ELISA. Nuclear factor of activated T cell 5 (NFAT5) expression was knocked down with siRNA. High extracellular NaCl induced NLRP3 and pro-IL-1β gene expression, while the gene expression of further inflammasome-associated proteins (NLRP1, NLRP2, NLRP6, NLRP7, NLRP12, NLRC4, AIM2, ASC, procaspase-1, pro-IL-18) was not altered or below the detection threshold. The NaCl-induced NLRP3 gene expression was partially dependent on the activities of phospholipase C, IP_3_ receptors, protein kinase C, the serum and glucocorticoid-regulated kinase, p38 MAPK, ERK1/2, JNK, PI3K, and the transcription factors HIF-1 and NFAT5. Pannexin-dependent ATP release and P2Y_1_ receptor activation is required for the full induction of NLRP3 gene expression. High NaCl induced a transient increase of the NLRP3 protein level and a moderate NLRP3 inflammasome activation, as indicated by the transient increase of the cytosolic level of mature IL-1β. High NaCl also induced secretion of IL-18.

**Conclusion:**

High extracellular NaCl induces priming of the NLRP3 inflammasome in RPE cells, in part via P2Y_1_ receptor signaling. The inflammasome priming effect of NaCl suggests that high intake of dietary salt may promote local retinal inflammation implicated in the development of AMD.

## Introduction

Age-related macular degeneration (AMD) is the most common cause of irreversible blindness in the elderly of developed countries [1]. The majority of patients suffer from the dry form of AMD which is, in the late stage, characterized by geographic atrophy, i.e., degeneration of the outer retina including the retinal pigment epithelium (RPE). The remaining patients suffer from the neovascular form characterized by choroidal neovascularization. AMD is associated with systemic and local inflammation [2–4]. Generally, inflammatory processes are activated by cytosolic protein-signaling complexes, termed inflammasomes. Inflammasomes drive the proteolytic activation of caspase-1 and maturation of the inflammatory cytokines interleukin (IL)-1β and IL-18 [5, 6]. Inflammasomes are a group of protein complexes that consist of at least three components, (i) a receptor molecule that recognizes pathogen- and damage-associated molecular patterns, e.g., NOD (nucleotide-binding oligomerization domain receptors)-like receptors (NLRs), (ii) the adaptor protein ASC (apoptosis-associated speck-like protein containing a caspase-activating recruitment domain), and (iii) the cysteine protease caspase-1 [6]. Various lines of evidence indicate that the NLRP3 inflammasome is expressed in the RPE of eyes affected by geographic atrophy or neovascular AMD [7], that NLRP3 inflammasome activation in RPE cells is implicated in mediating RPE cell degeneration in geographic atrophy [8, 9], and that NLRP3 inflammasome activation in the RPE may promote neovascular AMD pathologies like RPE barrier breakdown and choroidal neovascularization [10]. The NLRP3 inflammasome in RPE cells can be activated by various factors and conditions that are suggested to be implicated in the pathogenesis of AMD such as complement factors, Alu RNA accumulation, lipofuscin-mediated photooxidative damage, peroxidized lipids, lysosomal destabilization, and overexpression of vascular endothelial growth factor (VEGF) [7–15].

In addition to advanced age, race, and genetic factors, lifestyle factors such as sun light exposure, cigarette smoking, and nutrition influence the risk of AMD. Furthermore, systemic hypertension is a risk factor of AMD [16–18]. The main condition that causes acute hypertension is increased extracellular osmolarity following intake of dietary salt (NaCl) [19, 20]. High extracellular NaCl and extracellular hyperosmolarity are known causes of systemic immune activation [21, 22]. In murine macrophages, inflammasomes are sensors of hyperosmotic stress [23]. Because elevated extracellular osmolarity and high extracellular NaCl induce the production and secretion of angiogenic factors like VEGF and basic fibroblast growth factor (bFGF) in RPE cells [24, 25], high salt intake may contribute to the progression of AMD towards the neovascular stage. However, it is not known whether high extracellular NaCl also induces priming and activation of inflammasomes in RPE cells. Therefore, we determined the effects of high extracellular NaCl on the gene expression of inflammasome-associated proteins in human RPE cells. We found that high NaCl induces priming and transient activation of the NLRP3 inflammasome in RPE cells. The NaCl-induced priming of the NLRP3 inflammasome is in part dependent on autocrine/paracrine P2Y_1_ receptor signaling and the transcriptional activities of hypoxia-inducible transcription factor 1 (HIF-1) and nuclear factor of activated T cell 5 (NFAT5).

## Materials and Methods

### Ethics Statement

The study followed the tenets of Declaration of Helsinki for the use of human subjects. The use of human material was approved by the Ethics Committee of the University of Leipzig (approval #745, 07/25/2011). Tissues were obtained with the written informed consent from relatives of all donors.

### Materials

Cell culture components and solutions were purchased from Gibco BRL (Paisley, UK). The recombinant human IL-1 receptor antagonist was from R&D Systems (Abingdon, UK). AG1478, 8-cyclopentyl-1,3-dipropylxanthine (DPCPX), HIF inhibitor, LY294002, PD98059, SP600125, and SU1498 were obtained from Calbiochem (Bad Soden, Germany). A-438079, AR-C 118925XX, ARL-67156, 8-(3-chlorostyryl) caffeine (CSC), GSK650394, caffeic acid phenethyl ester (CAPE), MRS2179, the pannexin-blocking peptide ^10^panx, the scrambled control peptide ^10^panxScr, and SB203580 were from Tocris (Ellisville, MO). Ac-YVAD-CHO was obtained from Santa Cruz Biotechnology (Heidelberg, Germany), and L-leucyl-L-leucine methyl ester was from Bachem (Weil am Rhein, Germany). Stattic was from Enzo Life Science (Plymouth Meeting, PA), and PD173074 was kindly provided by Pfizer (Karlsruhe, Germany). Human-specific small interfering RNA (siRNA) against NFAT5 and nontargeted control siRNA were obtained from Qiagen (Hilden, Germany). AG1296, 2-aminoethoxydiphenyl borate (2-APB), apyrase, Gö6976, H-89, N-nitrobenzylthioinosine (NBTI), PP2, SB431542, U73122, and all other agents used were from Sigma-Aldrich (Taufkirchen, Germany), unless stated otherwise. The following antibodies were used: a mouse anti-human NLRP3 (1:750; Enzo, Lausen, Switzerland) a rabbit anti-human β-actin (1:1000; Cell Signaling, Frankfurt/M., Germany), anti-rabbit IgG conjugated with alkaline phosphatase (1:2000; Cell Signaling), and anti-mouse IgG conjugated with alkaline phosphatase (1:2000; Cell Signaling).

### Cell culture

The study followed the tenets of the Declaration of Helsinki for research involving human subjects. The use of human material was approved by the Ethics Committee of the University of Leipzig (#745, 07/25/2011). Post-mortem eyes from human cornea donors without reported eye disease were obtained within 48 h of death with the written informed consent from the relatives for the use of retinal tissue in basic science. RPE cells were prepared and cultured as described [26]. Cell lines derived from different donors were used in passages 3 to 5. In most experiments, near-confluent cultures (confluency ~90%) were growth arrested in medium without serum for 16 h, and subsequently, serum-free media with and without test substances were added. To determine whether the confluency degree of cultured RPE cells influences the high NaCl-induced gene expression of inflammasome-associated proteins and to examine the effect of NFAT5 siRNA, we used confluent cultures. The confluency of the cultures was evaluated microscopically. The isoosmotic control medium contained 127.6 mM NaCl and had an osmolarity of 281 mOsm/kg H_2_O. Hyperosmotic media were made up by addition of NaCl or sucrose. Addition of 50 and 100 mM NaCl to the culture medium resulted in osmolarities of the media of 381 and 457 mOsm/kg H_2_O, respectively. The hypoosmotic medium (60% osmolarity) was made up by adding distilled water. The cells were preincubated with pharmacological inhibitors for 30 min before osmotic challenge.

### RNA extraction and cDNA synthesis

Total RNA was extracted with the InviTrap Spin Universal RNA Mini Kit (Stratec Molecular, Berlin, Germany). The quality of the RNA was analyzed by agarose gel electrophoresis. The A_260_/A_280_ ratio of the optical density was measured using the NanoDrop1000 device (peQLab, Erlangen, Germany), and was between 1.95 and 2.03 for all RNA samples, indicating sufficient quality. After treatment with DNase I (Roche, Mannheim, Germany), cDNA was synthesized from 1 μg total RNA using the RevertAid H Minus First Strand cDNA Synthesis kit (Fermentas, St. Leon-Roth, Germany).

### Real-time RT-PCR

Real-time RT-PCR was performed with the Single-Color Real-Time PCR Detection System (BioRad, Munich, Germany) using the primer pairs described in Table 1. The PCR solution contained 1 μl cDNA, specific primer set (0.2 μM each), and 7.5 μl of a 2x mastermix (iQ SYBR Green Supermix; BioRad) in a final volume of 15 μl. The following conditions were used: initial denaturation and enzyme activation (one cycle at 95°C for 3 min); denaturation, amplification and quantification, 45 cycles at 95°C for 30 s, 58°C for 20 s, and 72°C for 45 s; melting curve, 55°C with the temperature gradually (0.5°C) increased up to 95°C. The amplified samples were analyzed by standard agarose gel electrophoresis. The mRNA expression was normalized to the level of ß-actin mRNA. The changes in mRNA expression were calculated according to the 2^-Δ ΔCT^ method (CT, cycle threshold), with ΔCT = CTtarget gene − CT_actb_ and ΔΔCT = ΔCT_treatment_ − ΔCT_control_.

**Table 1:** Primer pairs used in PCR experiments.

Gene	Accession Number	Sense Primer Sequence	Antisense Primer Sequence	bp
ACTB	NM_001101	ATGGCCACGGCTGCTTCCAGC	CATGGTGGTGCCGCCAGACAG	237
GAPDH	NM_002046	GCAGGGGGGAGCCAAAAGGGT	TGGGTGGCAGTGATGGCATGG	219
VEGFA_188, 164, 120_	NM_003376.5, NM_001287044.1, NM_001025370.2	CCTGGTGGACATCTTCCAGGAGTA	CTCACCGCCTCGGCTTGTCACA	479, 407, 275
bFGF	NM_002006	AGAGCGACCCTCACATCAAG	ACTGCCCAGTTCGTTTCAGT	234
IL1B	NM_000576	GGGCCTCAAGGAAAAGAATC	TTCTGCTTGAGAGGTGCTGA	205
IL18	NM_001243211.1	AATGCACCCCGGACCATATTT	CCTGGGACACTTCTCTGAAAGA	203
NLRP1	NM_033007.3	AATGGCCTCTGGATGAAACGT	CTCTCACAGAAGGCTCCCATG	168
NLRP2	NM_001174083.1	ACAACTTTGAGACACCCCAAGT	TTCACCCCTGTATTCCCAATGG	161
NALP3	NM_183395.2	AGACAGCATTGAAGAGGAGTGG	TTTGTTGAGGCTCACACTCTCA	169
NLRP6	NM_001276700.1	CCTGCTTTTCATCACCAGCG	AGCTCTGGTCGATGAACTGGT	248
NLRP7	NM_001127255.1	CAAAGCCAGGTGAAAAGGAAGG	CTGTCCAGTCCAGCATACACTT	246
NLRP12	NM_144687.3	TTTGAGCGGATAAACAGGAAGGA	CTGGGGATCTTTTCTTGGAGTGA	150
NLRC4	NM_001302504.1	TGTCAAGTGAACCCTGTGACC	CTAGCACGTTCATCCTGTCGA	192
NRF2	NM_001145412	AGCTAGATAGTGCCCCTGGAA	GGTTTTCCGATGACCAGGACT	179
AIM2	NM_004833.1	GTGCTGCACCAAAAGTCTCTC	TGAAACATCTCCTGCTTGCCT	169
PYCARD (ASC)	NM_145182.2	CTGACGGATGAGCAGTACCAG	GGATGATTTGGTGGGATTGCC	222
CASP1	NM_033293.3	GCACACGTCTTGCTCTCATTATC	CCTTCCCGAATACCATGAGACAT	244
CASP4	NM_033306.2	GACAGCACAATGGGCTCTATCT	AGTCGTTCTATGGTGGGCATTT	152
CASP5	NM_001136110.1	TCGTGAAGAATTCCTGAGACTGT	CTGGCTGTGAGATTCTTTTCGTC	234
P2X7	NM_002562.5	AAGCTGTACCAGCGGAAAGA	GCTCTTGGCCTTCTGTTTTG	202
P2Y1	NM_002563	GGTAGGGAGGAGGAAGATGC	TAACTTTCCGACTGCGCTTT	174
P2Y2	NM_002564	CCACCTGCCTTCTCACTAGC	TGGGAAATCTCAAGGACTGG	163
A1 (ADORA1)	NM_000674	CCTCCATCTCAGCTTTCCAG	AGTAGGTCTGTGGCCCAATG	222
A2B (ADORA2B)	NM_000676	CTCCATCTTCAGCCTTCTGG	ACAAGGCAGCAGCTTTCATT	234

### Western blot analysis

The cells were were washed twice with prechilled phosphate-buffered saline (pH 7.4; Invitrogen, Paisley, UK), scraped into 80 μl of lysis buffer (50 mM Tris-HCl pH 8.0, 5 mM EDTA, 150 mM NaCl, 0.5% NP-40, 1% protease inhibitor cocktail), and agitated at 4°C for 30 min. Thereafter, the cell lysates were centrifuged at 13,000 x g for 10 min, and the supernatants were analyzed by immunoblotting. Equal amounts of protein were separated by 10% SDS-polyacrylamide gel electrophoresis; immunoreactive bands were visualized using 5-bromo-4-chloro-3-indolyl phosphate/nitro blue tetrazolium.

### ELISA

Cells were cultured at 3 x 10^3^ cells per well in 12-well plates. At a confluency of ~90%, the cells were cultured in serum-free medium for 16 h. Subsequently, the culture medium was changed, and the cells were stimulated with a hyperosmotic medium (+ 100 mM NaCl). Culture supernatants (1 ml) and cell lysates (150 μl) were collected after 3 and 6 h, and the levels of mature IL-1β and IL-18 were determined with ELISA (HSLB00C; R&D Systems; detection threshold: 0.067 pg/ml IL-1β; IL-18 ELISA Kit; RayBiotech, Norcross, GA).

### siRNA transfection

Cells were seeded at 7 x 10^4^ cells per well in 12-well culture plates and were allowed to growth up to confluency of 60–80%. Thereafter, the cells were transfected with NFAT5 siRNA and nontargeted siRNA, respectively (10 nM each), using HiPerfect reagent (Qiagen) in F-10 medium containing 10% fetal bovine serum (Invitrogen) according to the manufacturer's instructions. After 24 or 48 h, serum-free iso- or hyperosmotic medium (+ 100 mM NaCl) was added for 6 h. Total RNA was extracted, and the NLRP3 mRNA level was determined with real-time RT-PCR analysis.

### Cell viability

Cell viability was determined by trypan blue exclusion. Cells were seeded at 5 x 10^4^ cells per well in 6-well plates. After reaching a confluency of ~90%, the cells were cultured in serum-free medium for 16 h and then in serum-free iso- or hyperosmotic medium (+ 100 mM NaCl) for 2 and 6 h. After trypsinization, the cells were stained with trypan blue (0.4%), and the number of viable (non-stained) and dead (stained) cells were determined using a hemocytometer.

### Statistics

For each test, at least three independent experiments were performed using cell lines from different donors. Data are expressed as means ± SEM. Statistical analysis was made using Prism (Graphpad Software, San Diego, CA). Significance was determined by one-way ANOVA followed by Bonferroni's multiple comparison test and by Mann-Whitney *U* test, respectively, and was accepted at *P*<0.05.

## Results

### Gene expression of inflammasome-associated proteins

**Fig 1:**
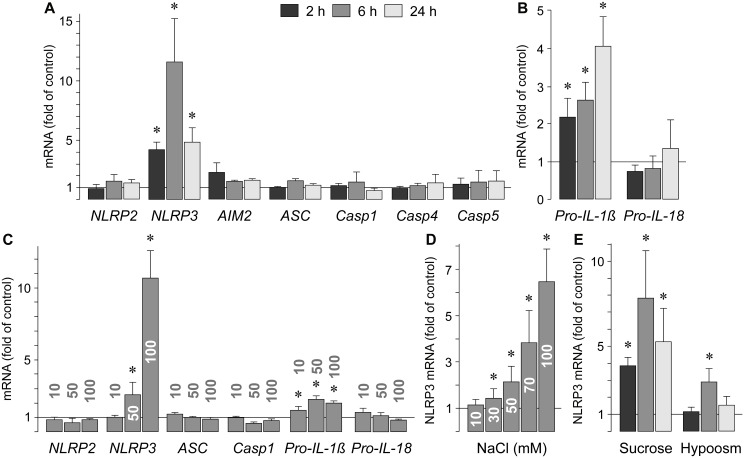
Osmotic regulation of the gene expression of inflammasome-associated proteins in human RPE cells. The mRNA levels were determined with real-time RT-PCR analysis in cells cultured 2, 6, and 24 h (as indicated by the panels of the bars) under hyper- and hypoosmotic conditions, respectively, and are expressed as folds of unstimulated control. Hyperosmolarity was induced by addition of NaCl (100 mM) or sucrose (200 mM) to the culture medium. Hypoosmolarity (60% osmolarity) was induced by addition of distilled water. **A.** Effects of high NaCl on the gene expression of inflammasome-associated proteins. *Casp*, caspase. **B.** Effects of high NaCl on the expression of pro-IL-1β and pro-IL-18 genes. **C.** Dose-dependent effects of high NaCl on the gene expression of inflammasome-associated proteins in confluent RPE cell cultures. 10, 50, and 100 mM NaCl were added to the culture medium, as indicated above the bars. **D.** Dose-dependent effect of high extracellular NaCl on the NLRP3 mRNA level in near-confluent cultures. 10 to 100 mM NaCl were added to the culture medium. **E.** Effects of sucrose-induced hyperosmolarity and extracellular hypoosmolarity on the expression of the NLRP3 gene. Each bar represents data obtained in 3–7 independent experiments using cell lines from different donors. Significant difference *vs*. unstimulated control: *P<0.05.

Activation of inflammasomes requires an initial priming signal and a subsequent activation signal [5, 6]. The priming event is triggered by signals which induce expression of inflammasome receptor and pro-IL-1β genes [5, 6]. To determine whether high extracellular NaCl induces expression of inflammasome-associated genes in RPE cells, we carried out real-time RT-PCR analysis using RNA extracted from cultured human RPE cell lines from different donors. Stimulation of the cells with high (+ 100 mM) extracellular NaCl induced a significant (*P*<0.05) increase in the cellular level of NLRP3 mRNA while the expression levels of other inflammasome receptor genes (NLRP2, AIM2) were not significantly (*P*>0.05) altered (Fig 1A). NLRP1, NLRP6, NLRP7, NLRP12, and NLRC4 mRNAs were not detected in RNAs extracted from different cell lines cultured under control and high NaCl conditions (not shown). Stimulation of RPE cells with high extracellular NaCl did also not alter the mRNA levels of the adaptor protein ASC and the inflammatory caspases 1, 4, and 5 (Fig 1A). High NaCl induced expression of the pro-IL-1β gene but not of the pro-IL-18 gene (Fig 1B). Similar results with upregulation of NLRP3 and pro-IL-1β gene expression, and no alterations in the expression of NLRP2, ASC, caspase 1, and pro-IL-18 genes, were found in confluent RPE cells stimulated with high NaCl (Fig 1C). The stimulatory effects on the expression of NLRP3 and pro-IL-1β genes suggest that high extracellular NaCl is a priming signal for the NLRP3 inflammasome in RPE cells.

The effect of high extracellular NaCl on the NLRP3 mRNA level was dose-dependent (Fig 1C and 1D). The NLRP3 mRNA level was also increased in cells cultured in media that were made up hyperosmotic by addition of 200 mM sucrose (Fig 1E) which induced an equal elevation of the extracellular osmolarity (by 200 mOsm/kg H_2_O) like 100 mM NaCl. The NaCl- (Fig 1A) and sucrose-induced expression of the NLRP3 gene (Fig 1E) displayed similar amplitudes and time dependencies, suggesting that the NaCl-induced NLRP3 gene expression was predominantly mediated by the elevation of the extracellular osmolarity. A hypoosmotic medium (60% osmolarity) induced a small transient increase of the NLRP3 gene expression (Fig 1E). The data indicate that the NLRP3 gene is transcriptionally activated in RPE cells by changes of the extracellular osmolarity.

### Intracellular signaling involved in NaCl-induced NLRP3 gene expression

**Fig 2:**
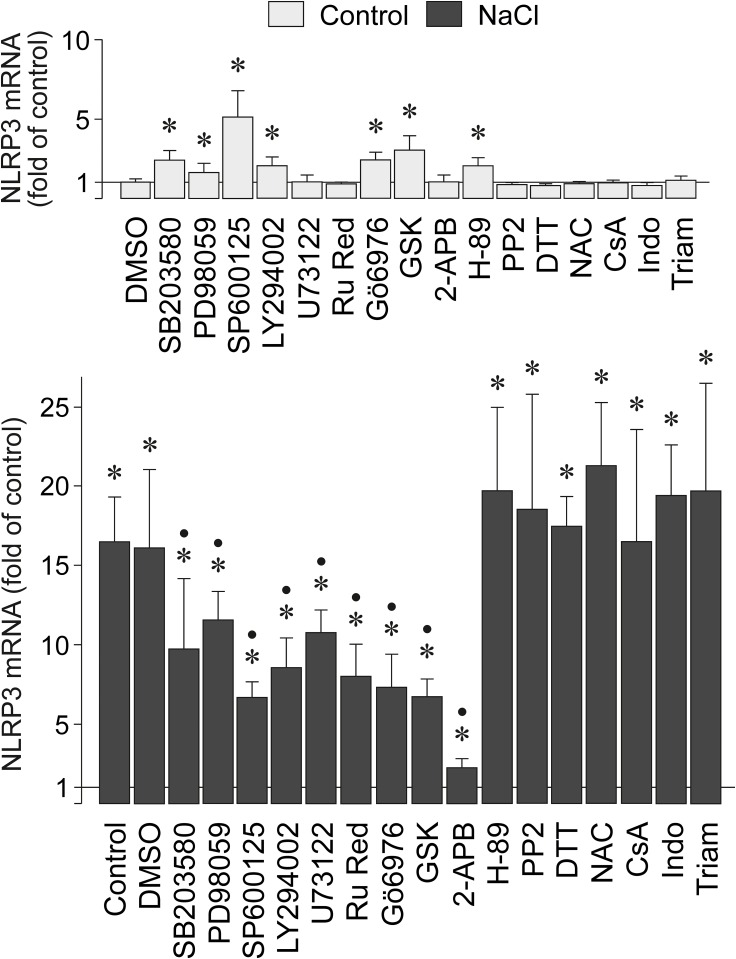
Intracellular signaling involved in the NaCl-induced expression of the NLRP3 gene in RPE cells. The level of NLRP3 mRNA was determined with real-time RT-PCR analysis in cells cultured 6 h in iso- (control) and hyperosmotic (+ 100 mM NaCl) media. The following agents were tested: the inhibitor of p38 MAPK activation, SB203580 (10 μM), the inhibitor of ERK1/2 activation, PD98059 (20 μM), the JNK inhibitor SP600125 (10 μM), the inhibitor of PI3K-related kinases, LY294002 (5 μM), the PLCγ inhibitor U73122 (4 μM), the inhibitor of calcium-binding proteins, ruthenium red (Ru Red; 30 μM), the inhibitor of PKCα/β, Gö6976 (1 μM), the SGK inhibitor GSK650394 (GSK; 1 μM), the inhibitor of store-operated calcium entry channels, IP_3_ receptors, and TRP channels, 2-APB (100 μM), the protein kinase A inhibitor H-89 (1 μM), the inhibitor of Src tyrosine kinases, PP2 (100 nM), the reducing agent dithiothreitol (DTT; 3 mM), the ROS inhibitor NAC (1 mM), the inhibitor of mitochondrial permeability transition, cyclosporin A (CsA; 1 μM), the cyclooxygenase inhibitor indomethacin (Indo; 10 μM), and the antiinflammatory glucocorticoid triamcinolone acetonide (Triam; 50 μM). Vehicle control was made with dimethylsulfoxide (DMSO; 1:1000). Means ± SEM of 3–8 independent experiments using cell lines from different donors. Significant difference *vs*. unstimulated control: *P<0.05. Significant difference *vs*. NaCl control: ●*P*<0.05.

To determine the intracellular signaling involved in mediating the NaCl-induced expression of the NLRP3 gene in RPE cells, we tested pharmacological blockers of key intracellular signal transduction molecules. The NaCl-induced NLRP3 gene expression was significantly (*P*<0.05) decreased, but not abrogated, by pharmacological inhibitors of the p38 mitogen-activated protein kinase (p38 MAPK; SB203580), extracellular signal-regulated kinases 1 and 2 (ERK1/2; PD98059), c-Jun NH_2_-terminal kinase (JNK; SP600125), and phosphatidylinositol-3 kinases (PI3K; LY294002) signal transduction pathways (Fig 2). The NaCl-induced expression of the NLRP3 gene was also decreased by inhibitors of phospholipase Cγ (PLCγ; U73122), calcium-binding proteins (ruthenium red), protein kinases C (PKC) α/β (Gö6976), and the serum and glucocorticoid-regulated kinase (SGK; GSK650394) (Fig 2). 2-APB, an inhibitor of store-operated calcium entry channels, inositol trisphosphate (IP_3_) receptors, and transient receptor potential (TRP) channels, fully prevented the NaCl-induced expression of the NLRP3 gene (Fig 2). The protein kinase A inhibitor H-89 and the inhibitor of Src tyrosine kinases PP2 had no effects (Fig 2). The cell-permeable reducing agent dithiothreitol and the reactive oxygen species (ROS) inhibitor N-acetyl-L-cysteine (NAC) did not inhibit the hyperosmotic expression of the NLRP3 gene (Fig 2). Furthermore, the inhibitor of mitochondrial permeability transition, cyclosporin A, was without effect (Fig 2). In addition, the cyclooxygenase inhibitor indomethacin and the antiinflammatory glucocorticoid triamcinolone acetonide did not inhibit the NaCl-induced NLRP3 gene expression (Fig 2). The data suggest that activation of various intracellular signal transduction cascades, as well as of PLC, IP_3_ receptors, PKC, and SGK are involved in mediating the stimulatory effect of high extracellular NaCl on the expression of the NLRP3 gene in RPE cells.

### Receptor-dependent signaling involved in NaCl-induced NLRP3 gene expression

It has been shown that extracellular hyperosmolarity induces a release of growth factors like VEGF, bFGF, and transforming growth factor (TGF)-β1 from RPE cells [24, 25]. To determine whether growth factor receptor signaling is involved in mediating the hyperosmotic expression of the NLRP3 gene in RPE cells, we tested pharmacological inhibitors of the following receptor kinases: VEGF receptor-2 (SU1498; 10 μM), platelet-derived growth factor receptor tyrosine kinase (AG1296; 10 μM), epidermal growth factor receptor tyrosine kinase (AG1478; 600 nM), TGF-β1 superfamily activin receptor-like kinase receptors (SB431542; 10 μM), and FGF receptor kinase (PD173074; 500 nM). Neither of these agents inhibited the increase of the NLRP3 gene expression in cells stimulated with high (+ 100 mM) NaCl for 6 h (data not schown).

**Fig 3:**
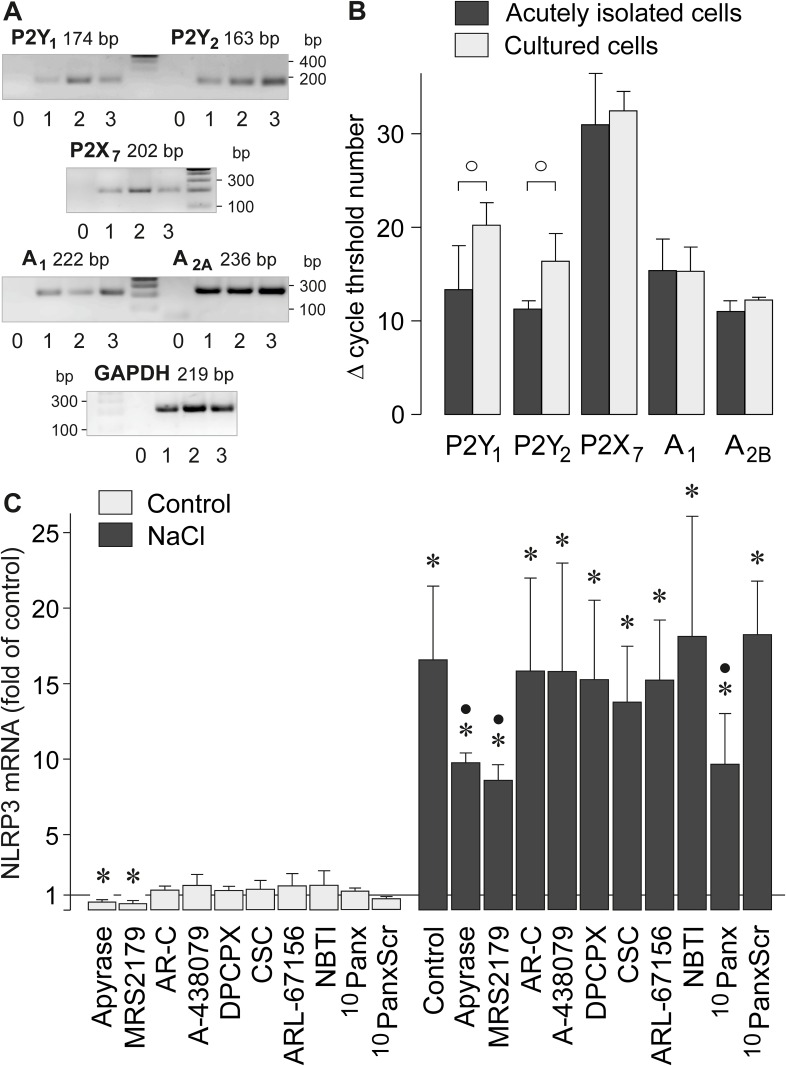
Involvement of P2Y_1_ receptor signaling in mediating the NaCl-induced expression of the NLRP3 gene in RPE cells. **A.** Presence of P2Y_1_, P2Y_2_, P2X_7_, adenosine A_1_ receptor, and adenosine A_2B_ receptor gene transcripts in the cells. To confirm the correct lengths of PCR products, agarose gel electrophoresis was carried out using products obtained from cultured cells of the 4th passage (1) and from cells that were acutely isolated from eyes of two post-mortem donors without apparent eye diseases (2, 3). Negative controls (0) were done by adding double-distilled water instead of cDNA as template. **B.** Relative expression levels of the receptor genes in acutely isolated and cultured cells, as revealed by real-time RT-PCR. Each bar represents the cycle number necessary for the detection of the transcript. **C.** The level of NLRP3 mRNA was determined with real-time RT-PCR analysis in cells cultured 6 h in iso- (control) and hyperosmotic (+ 100 mM NaCl) media. The following agents were tested: the ATP/ADP phosphohydrolase apyrase (10 U/ml), the P2Y_1_ receptor antagonist MRS2179 (30 μM), the P2Y_2_ receptor antagonist AR-C 118925XX (AR-C; 10 μM), the P2X_7_ receptor antagonist A-438079 (50 nM), the adenosine A_1_ receptor antagonist DPCPX (50 nM), the adenosine A_2A_ receptor antagonist CSC (200 nM), the ecto-ATPase inhibitor ARL-67156 (50 μM), the antagonist of nucleoside transporters, NBTI (10 μM), the pannexin-blocking peptide ^10^panx (200 μM), and the scrambled control peptide ^10^panxScr (200 μM). Means ± SEM of 3–6 independent experiments using cell lines from different donors. Significant difference *vs*. unstimulated control: *P<0.05. Significant difference *vs*. NaCl control: ●*P*<0.05. ○*P*<0.05.

Adenosine 5'-triphosphate (ATP) released from stressed cells is a danger signal which activates the NLRP3 inflammasome in various cell systems [27]. Extracellular ATP acts at purinergic metabotropic (P2Y) and ionotropic (P2X) receptors. RPE cells were shown to express multiple purinergic receptor subtypes including P2Y, P2X, and adenosine receptors [8, 28]. By using RT-PCR analysis, we found that both acutely isolated and cultured RPE cells contain transcripts of P2Y_1_, P2Y_2_, P2X_7_, and adenosine A_1_ and A_2B_ receptor genes (Fig 3A). The expression levels of P2X_7_, A_1_, and A_2B_ receptor genes were similar in acutely isolated and cultured cells while the expression levels of P2Y_1_ and P2Y_2_ receptor genes were lower in cultured cells compared to acutely isolated cells, as indicated by the significantly (*P*<0.05) increased cycle numbers necessary for the detection of the transcripts (Fig 3B). The expression levels of P2X_7_ transcipts in acutely isolated and cultured cells were very low, as indicated by the high cycle threshold numbers (Fig 3B).

To determine whether purinergic receptor signaling is involved in mediating the NaCl-induced NLRP3 gene expression in RPE cells, we used pharmacological receptor antagonists. We found that addition of the ATP-hydrolyzing enzyme apyrase and the P2Y_1_ receptor antagonist MRS2179, respectively, to the culture medium decreased significantly (*P*<0.05) the NLRP3 mRNA level in RPE cells under unstimulated control conditions (Fig 3C). Both agents also decreased (by 40–50%) the NaCl-induced NLRP3 gene expression (Fig 3C). On the other hand, antagonists of P2Y_2_ (AR-C 118925XX), P2X_7_ (A-438079), adenosine A_1_ (DPCPX), and adenosine A_2A_ receptors (CSC), as well as the ecto-ATPase inhibitor ARL-67156 and the antagonist of nucleoside transporters, NBTI, had no effects on the cellular level of NLRP3 transcripts under control and NaCl-stimulated conditions (Fig 3C). The data suggest that the NLRP3 gene expression under normal and high NaCl conditions is in part regulated by autocrine/paracrine purinergic signaling that involves a release of ATP and P2Y_1_ receptor activation. It has been shown that hyperosmotic stress induces a pannexin-1-mediated release of ATP from T cells [29]. We found that the pannexin-blocking peptide ^10^panx decreased the NaCl-induced expression of the NLRP3 gene in RPE cells to a similar extent like apyrase while a scrambled control peptide had no effect (Fig 3C). The data suggest that high extracellular NaCl induces a pannexin-dependent release of ATP from RPE cells.

### Role of transcription factors in NaCl-induced NLRP3 gene expression

**Fig 4:**
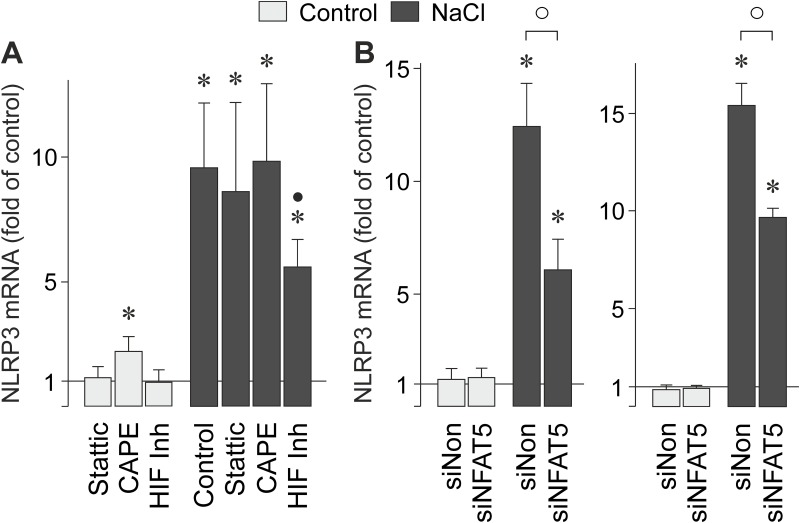
Involvement of transcription factor activities in the NaCl-induced expression of the NLRP3 gene in RPE cells. The mRNA level was determined with real-time RT-PCR analysis in cells cultured 6 h in iso- (control) and hyperosmotic (+ 100 mM NaCl) media. **A.** The following pharmacological agents were tested: the STAT3 inhibitor Stattic (1 μM), the NF-κB inhibitor CAPE (5 μM), and a HIF inhibitor (HIF-Inh; 5 μM). **B.** Effects of nontargeted siRNA (siNon) and NFAT5 siRNA (siNFAT5) on the level of NLRP3 mRNA. The cells were transfected for 24 h (non-confluent cultures; *left*) and 48 h (confluent cultures; *right*) before stimulation with high NaCl. Means ± SEM of 3–8 independent experiments using cell lines from different donors. Significant difference *vs*. unstimulated control: *P<0.05. Significant difference *vs*. NaCl control: ●*P*<0.05. ○*P*<0.05.

It has been shown that hyperosmotic stress induces expression of various transcription factors in RPE cells like HIF-1α, nuclear factor (NF)-κB, and NFAT5 [24]. To determine which transcription factors mediate the NaCl-induced NLRP3 gene expression in RPE cells, we used pharmacological blockers. The NaCl-induced NLRP3 gene expression was not altered in the presence of the inhibitor of signal transducer and activator of transcription 3 (STAT3), Stattic [30], and the NF-κB inhibitor CAPE [31], while a HIF inhibitor [32] partially inhibited the hyperosmotic expression of the NLRP3 gene (Fig 4A).

In various cell systems, cellular survival in hyperosmotic stress depends on the transcriptional activity of NFAT5 [33]. To determine whether NFAT5 activity is involved in mediating the NaCl-induced expression of the NLRP3 gene in RPE cells, we knocked down NFAT5 expression by transfection of the cells with NFAT5 siRNA. As negative control, nontargeted scrambled siRNA was used. As shown in Fig 4B, high NaCl induced a significantly (*P*<0.05) smaller increase of the NLRP3 mRNA level in cells transfected with NFAT5 siRNA compared to cells transfected with nontargeted siRNA. The data suggest that the NaCl-induced expression of the NLRP3 gene in RPE cells is in part dependent on the activities of HIF-1 and NFAT5.

### High NaCl induces transient NLRP3 inflammasome acivation

**Fig 5:**
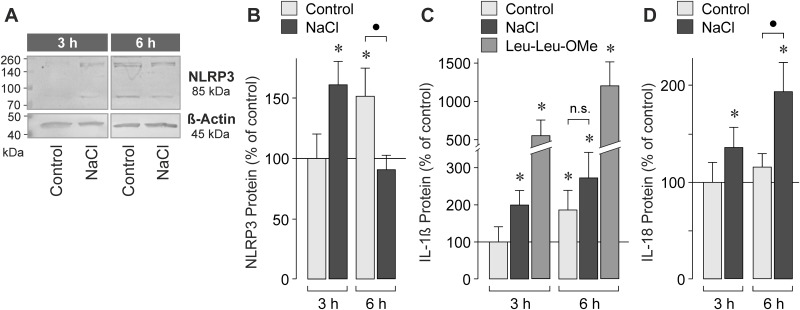
High extracellular NaCl induces transient activation of the NLRP3 inflammasome in RPE cells. The cells were cultured 3 and 6 h in isoosmotic (control) and hyperosmotic (+ 100 mM NaCl) media. **A.** Western blot analysis of the NLRP3 protein in cell lysates. Equal amounts of total protein (25 μg) were used for separation. **B.** Cytosolic level of the 85 kDa NLRP3 protein, as determined by densitometric analysis of Western blot data. The data were normalized to the level of β-actin protein, and are expressed as percent of unstimulated control measured after 3 h of stimulation (100%). **C.** Cytosolic level of mature IL-1β, as determined by ELISA. As positive control, NLRP3 inflammasome activation was induced by lysosomal destabilization with L-leucyl-L-leucine methyl ester (Leu-Leu-OMe; 1 mM). The data are expressed as percent of unstimulated control measured after 3 h of stimulation (100%; 1.02 ± 0.46 pg/ml). **D.** Level of IL-18 protein in the cultured media, as determined by ELISA. The data are expressed as percent of unstimulated control measured after 3 h of stimulation (100%; 0.19 ± 0.06 pg/ml). Means ± SEM of 3–7 independent experiments using cell lines from different donors. Significant difference *vs*. 3-h control: *P<0.05. ●*P*<0.05. n.s., not significant.

After the initial priming event, inflammasome activation is triggered by signals which induce assembly of NLRP3, ASC, and procaspase-1 into the inflammasome protein complex; activated inflammasomes drive the processing of procaspase-1 into the active form and the maturation of IL-1β [5, 6]. Western blot analysis showed that RPE cells contained NLRP3 protein at low level, and that the level of NLRP3 protein increased time-dependently under unstimulated control conditions (Fig 5A) (while the level of NLRP3 mRNA did not increase under control conditions; not shown). The data suggest that NLRP3 protein is continuously produced in unstimulated RPE cells. Stimulation with high extracellular NaCl induced a significant (*P*<0.05) increase of the cytosolic NLRP3 protein level after 3 h of stimulation, and a decrease of the NLRP3 protein level after 6 h of stimulation (Fig 5A and 5B).

In order to determine whether high extracellular NaCl induces activation of the NLRP3 inflammasome, we determined with ELISA the levels of mature IL-1β in RPE cell lysates and culture supernatants. As shown in Fig 5C, the cytosolic level of mature IL-1β increased time-dependently under unstimulated control conditions (while the level of pro-IL-1β mRNA did not increase under control conditions; not shown). High extracellular NaCl induced a significant (*P*<0.05) increase (to 199.9 ± 39.5% of control, 100%) of the cytosolic IL-1β level after 3 h of stimulation (Fig 5C). On the other hand, the cytosolic IL-1β level in cells cultured 6 h under control and NaCl-stimulated conditions were not significantly (*P*>0.05) different (Fig 5C). As positive control, lysosomes were destabilized using the lysosomotropic agent L-leucyl-L-leucine methyl ester which is known to induce NLRP3 inflammasome activation in RPE cells [7]. As shown in Fig 5C, lysosomal destabilization resulted in a time-dependent increase of the cytosolic level of mature IL-1β in RPE cells, in the mean to 555.6 ± 203.6% and 1205.8 ± 315.7% of control (100%), respectively, after 3 and 6 h of stimulation. However, we did not detect IL-1β with ELISA in the conditioned media of cells cultured 3 or 6 h under isoosmotic control and hyperosmotic (+ 100 mM NaCl) conditions, or in the presence of L-leucyl-L-leucine methyl ester (1 mM; data not shown). On the other hand, we found a time-dependent increase of the IL-18 protein level in the cultured media during stimulation of the cells with high extracellular NaCl (Fig 5D), suggesting that high NaCl induces a secretion of IL-18 from RPE cells. The data suggest that high NaCl induces a transient activation of the NLRP3 inflammasome resulting in maturation of IL-1β and secretion of IL-18.

### Involvement of P2Y_1_ receptor activation in NaCl-induced angiogenic factor expression

**Fig 6:**
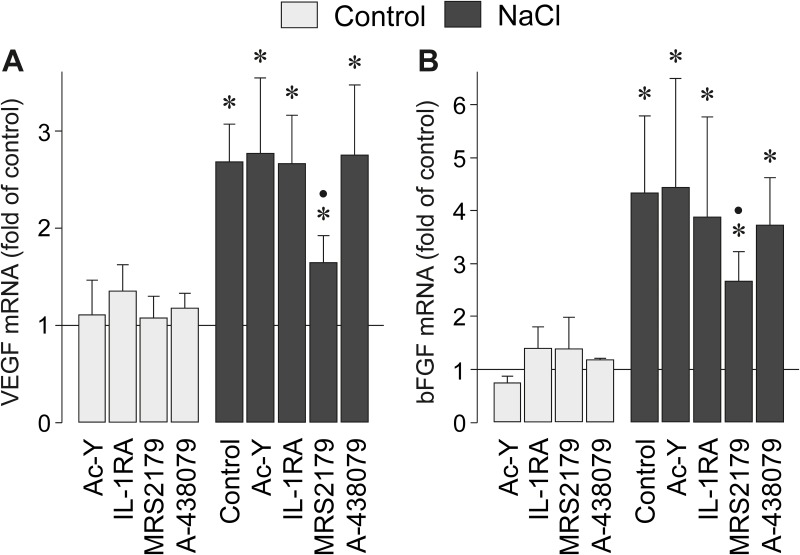
The NaCl-induced expression of VEGF (A) and bFGF (B) genes depends in part on P2Y_1_ receptor signaling. The mRNA levels were determined with real-time RT-PCR analysis in cells cultured 6 h in iso- (control) and hyperosmotic (+ 100 mM NaCl) media, and are expressed as folds of control. The following pharmacological agents were tested: the caspase-1 inhibitor Ac-YVAD-CHO (Ac-Y; 500 nM), a recombinant human IL-1 receptor antagonist (IL-1RA; 1 μg/ml), the P2Y_1_ receptor antagonist MRS2179 (30 μM), and the P2X_7_ receptor antagonist A-438079 (50 nM). Means ± SEM of 3–7 independent experiments using cell lines from different donors. Significant difference *vs*. unstimulated control: *P<0.05. Significant difference *vs*. NaCl control: ●*P*<0.05.

It has been shown that high extracellular NaCl induces gene expression of angiogenic factors like VEGF and bFGF in RPE cells [24, 25]. The caspase-1 inhibitor Ac-YVAD-CHO and a recombinant human IL-1 receptor antagonist did not inhibit the NaCl-induced expression of VEGF and bFGF genes in RPE cells (Fig 6A and 6B), suggesting that the hyperosmotic expression of angiogenic factor genes is independent on inflammasome activation. However, the NaCl-induced expression of VEGF and bFGF genes was significantly (*P*<0.05) decreased by the P2Y_1_ receptor antagonist MRS2179 while the P2X_7_ receptor antagonist A-438079 had no effect (Fig 6A and 6B).

### RPE cell viability

**Fig 7:**
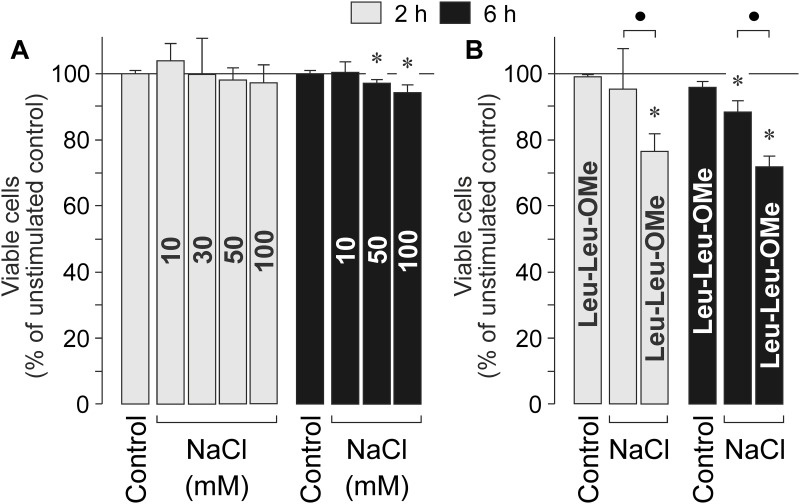
NaCl-induced alteration of the RPE cell viability. Data were obtained after 2 and 6 h of stimulation with high NaCl and are expressed as percent of unstimulated control (100%). **A.** Dose-dependency of the RPE cell viability. The doses of NaCl (in mM) added to the culture medium are given in the bars. **B.** Influence of lysosomal destabilization with L-leucyl-L-leucine methyl ester (Leu-Leu-OMe; 1 mM) on the RPE cell viability in the absence and presence of high (+ 100 mM) NaCl. Each bar represents data obtained in 5 independent experiments using cell lines from different donors. Significant difference *vs*. unstimulated control: *P<0.05. ●*P*<0.05.

To determine whether osmotic stress alters RPE cell viability, we measured the viability of cells cultured in the presence of different NaCl concentrations. As shown in Fig 7A, increases of the extracellular NaCl level by more than 10 mM induced a moderate, dose-dependent decrease in the viability of RPE cells which was significant (*P*<0.05) after 6 h of stimulation. Induction of NLRP3 inflammasome activation by lysosomal destabilization with L-leucyl-L-leucine methyl ester resulted in a significant (*P*<0.05) decrease of the cell viability in the presence but not in the absence of high NaCl (Fig 7B).

## Discussion

Chronic innate immune activation contributes to the dysfunction and progressive degeneration of the RPE underlying AMD [2, 4]. Activation of the NLRP3 inflammasome was implicated in mediating the degeneration of the RPE in geographic atrophy [8, 9], the late stage of dry AMD, and the development of choroidal neovascularization [7, 10], the hallmark of neovascular AMD. Systemic hypertension is a risk factor of AMD [16–18]. The main condition that induces acute hypertension is the increase of the extracellular osmolarity following intake of dietary salt [19, 20]. Elevated extracellular osmolarity and high extracellular NaCl are known to induce systemic immune activation [21, 22]. In the present study, we investigated whether high extracellular osmolarity and NaCl induce priming and activation of inflammasomes in RPE cells. We found that high extracellular NaCl induces expression of the NLRP3 gene in RPE cells (Fig 1A) while the expression of other inflammasome receptor genes remained unaltered (NLRP2, AIM2) or was below the detection threshold (NLRP1, NLRP6, NLRP7, NLRP12, NLRC4). High NaCl also induced expression of the pro-IL-1β gene and had no effect on the pro-IL-18 gene expression (Fig 1B). The data are consistent with recent studies which showed that IL-18, but not IL-1β, is constitutively expressed in RPE cells [14], and that NLRP3 inflammasome activation in a RPE cell line results in production of IL-1β, but not IL-18 [34]. The findings that high NaCl did not induce expression of ASC and procaspase-1 genes (Fig 1A) are in line with a study in macrophages which showed that lipopolysaccharide-induced priming of the cells involves NLRP3 and pro-IL-1β gene expression, but not alterations of ASC and procaspase-1 gene expression [35]. The stimulatory effects on the expression of NLRP3 and pro-IL-1β genes suggest that high extracellular NaCl induces priming of the NLRP3 inflammasome in RPE cells.

The increases of the levels of NLRP3 and mature IL-1β proteins (Fig 5A–5C) indicate that the NLRP3 inflammasome is continuously activated in cultured RPE cells under unstimulated control conditions (Fig 5A–5C). Priming of the NLRP3 inflammasome under unstimulated control conditions is also suggested by the effects of apyrase and the P2Y_1_ receptor antagonist which decreased the control level of NLRP3 gene expression (Fig 3C). High extracellular NaCl induced a transient increase of the NLRP3 protein level in RPE cells (Fig 5A and 5B) and a transient activation of the NLRP3 inflammasome, as indicated by the increase of the cytosolic level of mature IL-1β which was observed after 3 h of stimulation (Fig 5C). After 6 h of stimulation with high NaCl, the NLRP3 protein level returned to the control level, and the cytosolic IL-1β level was not different between cells cultured under control and high-NaCl conditions (Fig 5B and 5C). On the other hand, continuous activation of the NLRP3 inflammasome induced by lysosomal destabilization with L-leucyl-L-leucine methyl ester resulted in a higher level of cytosolic IL-1β after 6 h compared to 3 h of stimulation (Fig 5C). We did not detect IL-1β in the conditioned media of cells cultured in the presence of high NaCl or L-leucyl-L-leucine methyl ester (not shown). However, we found that high NaCl induces a secretion of IL-18 from RPE cells (Fig 5D). The present data are in agreement with a recent study which showed that RPE cells (in contrast to bone marrow-derived cells) release IL-18 rather than IL-1β in response to various triggers of inflammasome activation [14]. We found that the activation of the NLRP3 inflammasome is transient under high-NaCl conditions. It can not be ruled out that the time-dependent deactivation of the NLRP3 inflammasome and the failure of IL-1β secretion result (at least in part) from the reduction of the cell viability under high NaCl conditions (Fig 7A). However, the fact that activation of the NLRP3 inflammasome by lysosomal destabilization decreases the RPE cell viability (Fig 7B) may suggest that the time-dependent deactivation of the NLRP3 inflammasome supports the survival of RPE cells under high NaCl conditions.

We found that both extracellular hyper- and hypoosmolarity induced NLRP3 gene expression (Fig 1A and 1E). This is consistent with previous studies which showed that various activators of the NLRP3 inflammasome produce cell shrinking or swelling which can be also induced by extracellular hyper- and hypoosmolarity, respectively [36, 37]. However, the hyper- and hypoosmotic expression of the NLRP3 gene in RPE cells is likely mediated by different signal transduction mechanisms. We found that the activity of NFAT5 is involved in mediating the hyperosmotic induction of the NLRP3 gene expression (Fig 4B). It has been shown that the expression of NFAT5 in RPE cells is increased by extracellular hyperosmolarity and decreased by extracellular hypoosmolarity [24]. The lack of NFAT5 activation under hypoosmotic conditions may (at least in part) explain the relatively small activation of the NLRP3 gene under these conditions (Fig 1E). The data also suggest that different intracellular signaling mechanisms may contribute independently to the NaCl-induced expression of the NLRP3 gene.

We found evidence that multiple signal transduction pathways mediate the NaCl-induced expression of the NLRP3 gene in RPE cells. The NaCl-induced NLRP3 gene expression is in part dependent on activation of p38 MAPK, ERK1/2, JNK, PI3K signal transduction pathways, and the activities of phospholipase C, IP_3_ receptors, protein kinase C, and SGK. SGK, a main mediator of cellular sodium homeostasis, is induced by high extracellular NaCl in various cell systems and increases the protein abundance and activity of ion channels, carriers, and the sodium/potassium-ATPase in response to osmotic stress [38, 39]. We also found evidence that the NaCl-induced expression of the NLRP3 gene depends in part on the transcriptional activities of HIF-1 and NFAT5. However, it remains to be determined whether the NLRP3 gene is directly or indirectly activated by HIF-1 and NFAT5. It is likely that the transcriptional activities of HIF-1 and NFAT5 mediate the expression of protein kinases like SGK [40] which are involved in the regulation of the RPE cell response to osmotic stress. The present data are in agreement with previous studies which showed in various cell systems that HIF-1 activity is implicated in the induction of NLRP3 inflammasome activation [41, 42].

It has been shown that the NLRP3 inflammasome can be activated by ROS-dependent and -independent mechanisms [43]. We found that the reducing agent dithiothreitol and the ROS inhibitor NAC did not alter the NaCl-induced expression of the NLRP3 gene (Fig 2). The data suggest that oxidative stress does not play a role in the NaCl-induced priming of the NLRP3 inflammasome in RPE cells. In addition, the inhibitor of mitochondrial destabilization, cyclosporin A, did not reduce the NaCl-induced expression of the NLRP3 gene (Fig 2), suggesting that mitochondrial dysfunction, a main cause of cellular oxidative stress, plays no role. The data are consistent with our observations that high NaCl does not induce gene expression of the nuclear factor E2-related factor-2 (NRF2) in RPE cells (not shown), a key transcription factor that regulates the cellular antioxidative defense, and that the high NaCl-induced expression of the NFAT5 gene is also not dependent on oxidative stress and mitochondrial dysfunction (not shown).

We found evidence that autocrine/paracrine purinergic signaling is required for the full expression of the NLRP3 gene in response to osmotic stress. This signaling involves a pannexin-dependent release of ATP and P2Y_1_ receptor activation (Fig 3C). Activation of P2Y_1_ receptors may trigger a PLC- and IP_3_-mediated calcium mobilization from internal stores and activation of PKC; inhibition of these intracellular pathways decreased the NaCl-induced expression of the NLRP3 gene (Fig 2) to a similar extent as apyrase and the P2Y_1_ receptor blocker MRS2179 (Fig 3C). PLC- and IP_3_-mediated calcium mobilization was described to be critical for the activation of the NLRP3 inflammasome in macrophages after stimulation with extracellular ATP [44]. We also found that the NaCl-induced expression of VEGF and bFGF genes depends in part on P2Y_1_ receptor signaling (Fig 6A and 6B). However, because the NaCl-induced expression of VEGF and bFGF genes was independent on inflammasome activation (Fig 6A and 6B), activation of P2Y_1_ receptors may independently trigger angiogenic factor expression and priming of the inflammasome in RPE cells. The finding that the NaCl-induced expression of VEGF and bFGF genes is independent on inflammasome activation is in line with a recent study which showed that inflammasome activation in RPE cells reduces, but not increases, the constitutive secretion of VEGF [45].

We found significant effects of high extracellular NaCl on the expression of the NLRP3 gene when more than 10 mM NaCl were added to the culture medium (Fig 1D). It is generally accepted that the highest pathological blood osmolarity in human subjects is around 360 mOsm/kg which can be achieved by addition of 40 mM NaCl to the culture medium [39, 46]. However, less well appreciated is that the local extracellular NaCl concentration in the interstitium may be considerably higher (160–250 mM) than the plasma concentration of NaCl (~140 mM) [47, 48]. Therefore, the present results may have relevance for *in-vivo* conditions.

In the developed world, the intake of dietary salt rapidly increased in the past along with the consumption of processed foods which often contain salt contents more than 100 times higher compared to home-made meals [49]. Increased intake of dietary salt may represent an environmental risk factor for the progression of AMD [24]. Here, we show that high extracellular NaCl induces priming of the NLRP3 inflammasome in RPE cells. In addition to the stimulatory effects of high salt on systemic immune processes [21, 22], salt-induced inflammasome priming in RPE cells may contribute to retinal inflammation in AMD. High salt may render RPE cells more susceptible to inflammatory stimuli and pathogenic factors involved in mediating degeneration of the RPE. This assumption is supported by the fact that lysosomal destabilization induces RPE cell death under high NaCl conditions but not under control conditions (Fig 7B). This mechanism may link dietary salt intake as the main cause of acute hypertension and innate immune activation in RPE pathology. Because the NLRP3 inflammasome is time-dependently deactivated in response to long-term NaCl stimulation (Fig 5A–5C), repetitive increases of the plasma NaCl level in postprandial phases will have greater effects than a persistent elevation of the NaCl level. We found that autocrine/paracrine P2Y_1_ receptor signaling is involved in mediating the salt-induced priming of the NLRP3 inflammasome in RPE cells and the expression of angiogenic factor genes. Because both effects may contribute to the development and progression of AMD, P2Y_1_ receptors may represent a target for the development of pharmacological approaches to treat age-related retinal inflammation and degeneration.
